# Age-dependent shaping of the social environment in a long-lived seabird: a quantitative genetic approach

**DOI:** 10.1098/rstb.2022.0465

**Published:** 2024-10-28

**Authors:** Maria Moiron, Sandra Bouwhuis

**Affiliations:** ^1^ Institute of Avian Research, Wilhelmshaven 26386, Germany; ^2^ Department of Evolutionary Biology, Bielefeld University, Bielefeld 33501, Germany

**Keywords:** ageing, density, phenotypic plasticity, social ageing, social behaviour, spatial distribution

## Abstract

Individual differences in social behaviour can result in fine-scale variation in spatial distribution and, hence, in the social environment experienced. Given the expected fitness consequences associated with differences in social environments, it is imperative to understand the factors that shape them. One potential such factor is age. Age-specific social behaviour—often referred to as ‘social ageing’—has only recently attracted attention, requiring more empirical work across taxa. Here, we use 29 years of longitudinal data collected in a pedigreed population of long-lived, colonially breeding common terns (*Sterna hirundo*) to investigate sources of variation in, and quantitative genetic underpinnings of, an aspect of social ageing: the shaping of the social environment experienced, using the number of neighbours during breeding as a proxy. Our analyses reveal age-specific declines in the number of neighbours during breeding, as well as selective disappearance of individuals with a high number of neighbours. Moreover, we find this social trait, as well as individual variation in the slope of its age-specific decline, to be heritable. These results suggest that social ageing might underpin part of the variation in the overall multicausal ageing phenotype, as well as undergo microevolution, highlighting the potential role of social ageing as a facilitator for, or constraint of, the evolutionary potential of natural populations.

This article is part of the discussion meeting issue ‘Understanding age and society using natural populations’.

## 1. Introduction

The social environment experienced by individuals can strongly affect their behaviour and fitness, such that social environment effects should be central tenets in ecological and evolutionary research. As such, ecological models of density dependence emphasize the effect of density on reproductive rates, survival and population size [1–4], and evolutionary models of social selection incorporate intraspecific interactions via resource competition [5–9]. Although most research is focused on population-level metrics of the social environment (i.e. population size or density), variation in the spatial distribution of individuals within populations can result in fine-scale among-individual variation in the social environment experienced [10–12]. Such variation might itself be driven by differences in social behaviour, such that it is important to quantify individual variation in social behaviour and its underlying drivers.

One potential factor driving individual differences in social behaviour is age. Longitudinal studies of various vertebrate populations increasingly provide evidence for age specificity of phenotypic traits [13–15], some of which represent social behaviour, with the resulting pattern being referred to as ‘social ageing’ ([16–18] and reviewed in Woodman *et al*. [19] (this issue)). The age-specific in- and decrease in social dominance in a resource acquisition setting in jackdaws (*Coloeus monedula*) provides a clear example of a direct change in social behaviour with age [20]. Social ageing, however, may not solely arise owing to direct changes in individual social behaviour with age, but may also indirectly result from age-specific changes in non-social behaviour (see Hasenjager *et al*. [21] for a discussion on knowledge transmission across age classes, and Harrison *et al*.[22] (this issue) for a review on social ageing in non-social species), space-use and demographic processes (see Gamelon *et al*. [23] (this issue), for an extended overview on demographic, behavioural and evolutionary processes). As an example, older birds often breed earlier in the season than younger birds [24], which may lead to them experiencing a reduced density, which in turn may reduce their (opportunity for) social interaction. As such, social ageing can be a highly complex process, the understanding of which requires investigating the dynamic links between age-dependent effects, demography and (social and non-social) behaviours to explore their relative contributions [18]. In addition, a comprehensive understanding of age-dependent changes in social behaviour, or the social environment experienced, will also require the investigation of patterns at different levels of variation, given that they can potentially occur at the population, individual and/or additive genetic level.

At the population level, longitudinal data are required to disentangle between-individual effects of age—owing to selective (dis)appearance of individuals, or differences between cohorts—from within-individual changes in social behaviour with age [13,15,25]. At the individual level, longitudinal analyses also provide valuable information to the study of social ageing, in particular to individual variation in social ageing rates [26]. The observation of a significant within-individual change in social behaviour with age at the population level does not readily imply that all individuals in a population age in the same way. Indeed, across different phenotypic traits, ageing rates have been shown, to a varying extent, to differ among individuals (e.g. [26–33] and [34] for social ageing studies specifically, reviewed in [15]). Finally, at the genetic level, classic evolutionary theory postulates that additive genetic variance should increase with age [35]. This theoretical prediction, however, has received mixed empirical support from studies using longitudinal data from natural populations (reviewed in [36,37]). Although some quantitative genetic studies indeed provided evidence for heritable variation in ageing patterns (i.e. genotype (G) × Age interactions) [33,38], others did not find support [27,28], such that it remains an open question to what extent individual differences in rates of ageing, including social ageing, are underpinned by additive genetic variation.

Many empirical studies on social ageing indicate that older adults are less social than younger ones (reviewed in [18] and by Woodman *et al*. [19] (this issue), but see [39]). Female red deer (*Cervus elaphus*), for instance, had reduced home range areas, interacted with fewer individuals within their home ranges and shifted their locations to less populated areas as they aged [40,41]. Similarly, killer whales (*Orcinus orca*) became less central in their contact network while growing older [42], and ageing yellow-bellied marmots (*Marmota flaviventris*) reduced both their affiliative and agonistic social behaviours [43,44]. While there is strong evidence for within-individual changes in social behaviour with age, there is considerably less evidence for selective disappearance of individuals with certain social behaviour, that is, where more (or less) social individuals are more (or less) likely to die and, hence, disappear from the population (see [34] and Cook *et al*. [45] (this issue) for a study on age structure and selection on social behaviour, but see also [40,41]). Furthermore, it is also worth pointing out that the vast majority of studies of social ageing in vertebrates are limited to humans (meta-analysis: [46]), non-human group-living primates [16–18,34,47] and other mammals [18,40–44] and focus on traits such as connectivity and affiliative interactions quantified using social network analyses, as opposed to metrics based on space-use and the social environment experienced. Increasing the breadth of taxonomic groups and social behaviours will pave the way to unravel the full complexity of patterns of social ageing in natural populations, as well as the underlying drivers of those patterns. In addition, it is also important to increase the breadth of levels of variation under investigation—that is, to incorporate studies of social ageing at the genetic level—for a better understanding of the extent and magnitude of heritable variation underpinning differences in social ageing.

In this study, we use data from a long-term individual-based study on a colonially breeding seabird, the common tern (*Sterna hirundo*). Previous analyses of the birds in this colony showed that their phenology [48], reproductive performance and survival [48,49], as well as various aspects of their physiology [50–53], migratory behaviour [54], foraging [55] and offspring provisioning [56] behaviour, vary with age. In addition, it was shown that older birds breed closer to the colony’s edges than younger birds [57]. Here, we ask whether the latter, which likely reflects settlement behaviour, can be viewed as an aspect of social ageing and shape the social environment that the birds experience during breeding. Given that individual-level breeding density is an important aspect of this social environment [10–12], we use the number of active neighbours during breeding as our social trait. Applying a quantitative genetic framework to 29 years of longitudinal data comprising 4710 observations of 878 individual females and information on pedigreed relatedness, we investigate population, individual and genetic variation in the number of neighbours at the time of breeding. Our specific objectives are to test for (i) age-specific changes in social behaviour, separating between-individual and within-individual effects, as well as (ii) patterns of individual and additive genetic variation in (the age-specific plasticity of) this social trait (i.e. individual and heritable differences in social ageing). Finally, we assess to what extent effects are owing to age *per se* or to the above-mentioned age effects on phenology and spatial distribution within the colony.

## 2. Methods

### (a) Study system and data collection

Data were collected between 1993 and 2021 as part of a long-term study of a free-living, colonially breeding population of common terns. The colony site is located at the Banter See on the German North Sea coast (53°36′ N, 08°06′ E) and comprises an alignment of six identical artificial islands, each measuring 10.7 × 4.6 m. The islands are spaced 90 cm apart and are protected from flooding and rat predation by a 60 cm wall. Standardized data collection was initiated in 1992; since this time, all locally hatched birds have been marked with individually numbered subcutaneously injected transponders shortly prior to fledging, and the presence and reproductive performance of marked individuals have been annually monitored [58].

Common terns at the Banter See lay their first eggs in early May [59], right after they have arrived from their wintering grounds along the West African coast [60] in April [61], at intervals of 1–2 days [62]. Given that the colony is checked for new clutches every 2–3 days throughout the breeding season, we are able to determine the exact date of clutch initiation for each nest. We define laying date as the date of the appearance of the first egg laid by a female in her first clutch of the season (1 January = 1) [48].

As part of the standard protocol, incubating parents are automatically identified by their transponders using portable antennae placed around each nest, and chicks are ringed within 1–2 days of hatching, when still at the nest. The social pedigree was constructed using these field observations of social parents and their offspring. We assume the social pedigree to be a good approximation of the genetic pedigree, given the low levels of extra-pair paternity [63]. We pruned the initial pedigree to remove individuals that were either not phenotyped or not ancestors to phenotyped individuals, only retaining informative individuals with respect to our analysis. The pruned pedigree consisted of 1042 individuals and had a maximum depth of six generations, 403 paternities and 309 maternities.

### (b) Data selection

We used the number of active neighbours at the time of breeding as our social trait, capturing variation in the social environment that birds experience. This trait likely results from settlement behaviour and underpins variation in the level of competition for resources related to space-use within the breeding colony. The Banter See common tern population is an ideal system in which to study the factors influencing the number of active neighbours at the time of breeding for multiple reasons. First, given the particular set-up of the study population, being a closed breeding site of fixed area, we are able to identify and mark all nests produced in the colony and, hence, quantify the presence and number of all breeding pairs and their timing of breeding. Second, the colony site is a homogeneous man-made structure. As such, besides the presence of the walls bordering the islands, with the associated edge effects, we can exclude potential confounding effects owing to variation in the physical environment, such as landscape features. Third, population density has strongly fluctuated across the 29 years of the study period, with the number of breeding pairs increasing since 1992 and ranging between 90 and 740 (see electronic supplementary material, figure S1*a*,*b*). This fluctuation ensures extensive, naturally occurring variation in individuals’ social environments.

At the end of each breeding season, once the birds depart towards their wintering grounds, all nest positions are mapped by measuring their distances from the wall of one long and one narrow side of each island, leading to *x*- and *y*-coordinates [64]. For each transpondered female, we used these coordinates to quantify the number of actively breeding neighbours in a 2 m radius, assigning a neighbour the ‘active’ status if it initiated its clutch within a period of four weeks before the laying date of the focal bird. Four weeks represent the time that a common tern needs between laying its first egg and having its last egg hatch—that is, the time when a breeding adult is attending its nest most frequently and interacting with its neighbours. In our analyses, we used data from females only because we have previously shown that although male genetic and non-genetic components explain some phenotypic variation in laying date, these male effects are of a substantially smaller magnitude than female components [65]. As such, egg-laying date in common terns at the Banter See is mostly a sex-limited trait, with social effects expected to operate via the females [65]. We evaluated our decision of using a 2 m radius by repeating our models quantifying social behaviour as the number of active neighbours using radii of 1, 3 and 4 m. This sensitivity analysis showed that our results were robust to the radius chosen (see electronic supplementary material for details).

**Figure 1.**
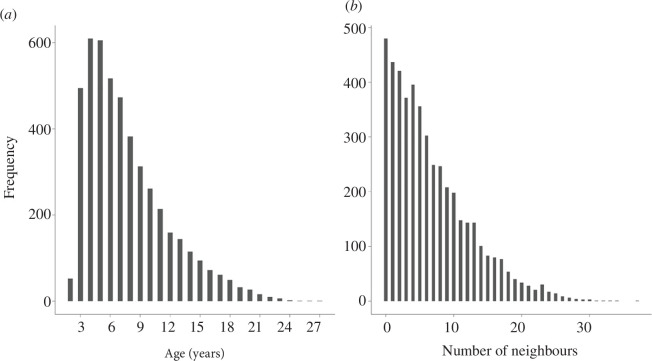
Histograms representing (*a*) the age distribution of the 878 female common terns included in the study and (*b*) the phenotypic distribution of the social environment experienced by these birds, measured as the number of active breeding neighbours in a 2 m radius from the focal female in a given year. An active neighbour is any bird that initiated its clutch four weeks before the laying date of the focal bird.

We tested for three variables to investigate patterns of phenotypic variation in the number of active neighbours. First, we used data on individual age, excluding individuals for which age was not known (i.e. excluding 181 observations on 22 females; 3.7%). Our data comprised observations from females aged 2–27 years, with a mean and s.d. of 7.67 ± 4.10 years (figure 1*a*). The average lifespan for adults of both sexes in the population is 7.4 years [66]. Second, we used data from a phenological trait closely linked to individual fitness in this species—egg-laying date—and data from a variable linked to the spatial distribution of females within the colony, the distance to the nearest colony wall. Overall, our data comprised 4710 observations from 878 individual females of known age and laying date, for which the number of active neighbours ranged between 0 and 37, with a mean and s.d. of 6.65 ± 5.79 (figure 1*b*).

### (c) Statistical analyses

#### (i) Temporal trend in neighbour density, age and laying date

We used the annual mean number of active neighbours to assess the temporal trend in our social trait. We did so by running a linear model with year as a covariate and the number of active neighbours as the response variable. The response variable was modelled assuming an overdispersed Poisson error distribution with log-link function (figure 1*b*) in this model and hereafter. We also tested for a temporal trend in age and laying date by running a linear model with age and laying date as the response variable, respectively, year as a covariate, and assuming a Gaussian error distribution.

#### (ii) Sources of variation in neighbour density

To understand the sources underpinning phenotypic variation in neighbour density, we built a univariate animal model where the number of active neighbours was the response variable. To test for social ageing at the population level, we modelled the effect of age. We applied a within-subject centring approach to partition this age effect into its between-individual and within-individual components (following [25]). As such, we split the age variable into (i) the mean age of each female and (ii) the female’s annual age deviation from her mean (delta age). The delta age term represents the within-individual change with age, and a significant effect would indicate social ageing, while a significant difference between the mean and delta age components would indicate selective (dis)appearance of females with certain social behaviour. A non-significant difference between the two age components, on the other hand, would indicate that within‐individual changes with age would be sufficient to explain the population-level age pattern [25]. We also modelled the interaction between the delta and mean age effects to test for nonlinearity of the within-individual age effect, that is, accelerating or decelerating social ageing.

**Figure 2.**
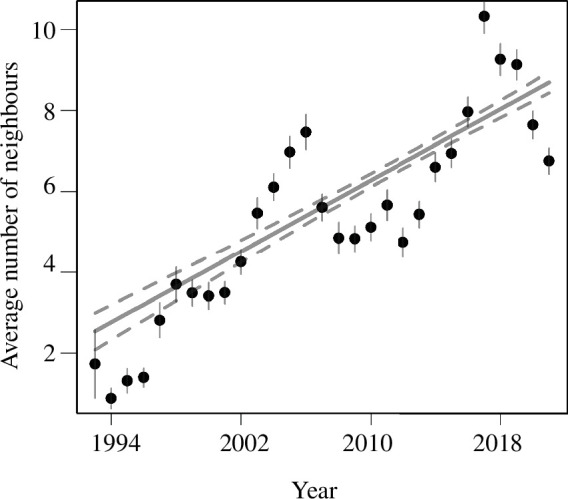
Temporal phenotypic trend in the number of actively breeding neighbours surrounding 878 focal female common terns across 29 years of study (1993–2021). Black dots and bars represent annual means with standard errors; grey solid and dashed lines represent the slope and associated 95% CI of the estimated temporal trend.

Additionally, we included three other fixed effects: year (as a continuous linear variable, to control for the temporal trend in the number of active neighbours; figure 2), colony density (as a continuous linear variable, ranging from 90 to 740 pairs; electronic supplementary material, figure S1*a*,*b*) and local (island) density at the time of breeding (measured as the number of nests initiated on the focal island in the four weeks before the egg-laying of the focal individual). We included the latter variable to control for the overall density differences among islands because within-colony, among-island differences in density could have potentially created spurious associations with the number of neighbours at the time of breeding, as individuals on average only nest at one or two islands in their lifetime (mean and s.d. number of islands used by a single individual = 1.60 ± 0.85, range = 1–5, individual repeatability = 0.88 [95% credible interval (CI) = 0.85, 0.90]; electronic supplementary material, figure S3). These three fixed effects were mean-centred and variance-standardized to facilitate the interpretation of their relative influence.

We included random intercepts for (i) individual identity linked to the pedigree to test for additive genetic effects, (ii) individual identity not linked to the pedigree to account for repeated measures and estimate the permanent environment effects, (iii) island identity to account for potential differences across the six islands (other than density) that constitute the overall colony, and (iv) year of breeding to control for variation among years. Repeatability (*R*) and heritability (*h*^2^), conditional to the variance explained by the fixed effects, were estimated as the proportion of the total phenotypic variance explained by the individual variance (i.e. sum of additive genetic and permanent environmental components) and additive genetic variance, respectively. Evolvability (CV_A_) was estimated as the coefficient of additive genetic variance [67,68].

After having run this model, we re-ran it while also including laying date and distance to the nearest wall (both fitted as mean-centred and variance-standardized continuous linear variables) to assess to what extent age effects (see §3) were owing to age *per se* or to known age effects on phenology [48] and/or distance to the wall [57]. Individuals that start breeding earlier and/or nest closer to the colony wall (potentially to reduce predation risk) might have fewer neighbours compared to those that start breeding later in the season when the colony is more crowded and/or in the centre of the islands where they may be less safe from aerial predators. In addition, to assess whether a finding of selective (dis)appearance of females with certain social behaviour was not owing to the inclusion of females that were still alive in our dataset, we re-ran our main model including only females that can be assumed to be dead because they have not been observed for two breeding seasons in a row [66]. To do so, we removed 1660 females that were observed in 2022 and/or 2023 (i.e. 35%). This re-analysis gave quantitatively very similar results (electronic supplementary material, table S2), indicating that our finding of selective disappearance (see §3) was robust.

#### (iii) Individual-by-age and genotype-by-age interactions

To model among-individual variation in social ageing (individual-by-age interactions, I × Age) using a random regression analysis [26], we added the interaction between individual identity and age to the model described above. In this model, we fitted the actual birds’ age as the gradient for the slopes rather than delta age. Whereas individuals were measured at different age ranges, and it would have been desirable to centre age within individuals, we found evidence for nonlinearity in the population-level plasticity of social behaviour (see §3), making actual age a more appropriate gradient. We mean-centred and variance-standardized age prior to fitting it as a gradient in the random slope model to ensure that the estimate of the intercepts’ variance is calculated for the average age [26,69], and slopes are expressed as the effects of a change of two standard deviations in age, facilitating cross-variable and cross-study comparisons. We fitted the same fixed effects as in the previous model without random slopes; however, we modelled actual age (mean-centred and variance-standardized) instead of the partitioned components of age (i.e. instead of the average and delta age components).

With this model, we tested for among-individual variance in the intercepts and slopes of social ageing while also testing for the covariance and correlation between individuals’ intercepts and slopes. Because inappropriate modelling of residual variance (e.g. assuming residual homogeneity) might lead to erroneous inferences of slope variance in random regression models [70], we accounted for potential changes in residual variance by fitting five, rather than one, residual variance components. We assumed uncorrelated residual variances.

Provided that a deviance information criterion (DIC) model comparison suggested that there was substantial individual variation in reaction norm slopes (I × Age; but see [71,72] for criticism on DIC model comparison), we further decomposed the individual variance component into its additive genetic (G) and permanent environment (PE) effects, the latter for example arising from differences in the developmental environment. To do so, we modelled the interactions between the additive genetic and permanent environmental variances with (mean-centred and variance-standardized) age as random effects (G × Age and PE × Age).

#### (iv) Statistical model implementation

All models were fitted using a Bayesian framework implemented in the statistical software R (v. 4.3.0) [73] using the package *MCMCglmm* [74]. We fitted parameter-expanded priors. Burn-in, total number of iterations and thinning intervals were chosen to ensure that all models achieved the targeted, effective sample size of 1000. The effective sample sizes yielded absolute autocorrelation values lower than 0.1 and satisfied convergence criteria based on the Heidelberger and Welch convergence diagnostic [75]. We drew inferences from posterior modes, medians and 95% credible intervals (95% CI).

## 3. Results

### (a) Temporal trend in population-level neighbour density, age and laying date

The overall mean (± s.d.) number of neighbours across the 29 years of data included in our study was 6.65 ± 5.79, and annual means significantly increased over the study period, with a rate of 0.22 neighbours per year (overall change = 6.16, 95% CI [5.53, 6.79]; figure 2).

Annual mean population age increased from 6 to 9 years, with some strong interannual fluctuations (*β* = 0.08 years per year, 95% CI [0.07, 0.10]; electronic supplementary material, figure S4), while mean laying date showed no change over the study period (*β* = 0.03 days per year, 95% CI [−0.02, 0.09]). Annual means of age and laying date were significantly correlated (*r* = −0.48, 95% CI [−0.72, −0.14]), with older birds breeding on average earlier than younger birds (electronic supplementary material, figure S5).

### (b) Sources of variation in neighbour density

Variation in the number of neighbours was explained by our age terms (table 1). Birds that were, on average, younger had a higher number of neighbours compared to birds that were, on average, older (main effect of mean age, posterior mode = −0.024, 95% CI [−0.032, −0.016]; table 1). Within individuals, birds also had fewer neighbours as they grew older (main effect of delta age, posterior mode = −0.059, 95% CI [−0.078, −0.039]; table 1), and the effect of this within‐subject component of age was greater than that of the between‐individual component (difference between within-individual and among-individual effects, posterior mode = 0.003, 95% CI [0.012, 0.054]). This latter finding provides evidence for selective disappearance, such that long-lived birds had a smaller number of neighbours and likely lower competition for access to resources throughout their lives. Additionally, we found evidence for nonlinearity of the within-individual age effect, with birds that were, on average, younger having a greater reduction in the number of neighbours with age (steep negative slope) than those that were, on average, older (effect of the interaction of delta age and mean age, posterior mode = 0.003, 95% CI [0.001, 0.005]; table 1). In other words, the within-individual decline in the number of active neighbours decelerated with age. Importantly, these observed age effects were independent of the strong positive association between the number of neighbours and local density at the time of breeding (table 1). Finally, there was no clear temporal trend in the number of neighbours over time (after correcting for all fixed effects) or effect of total colony size (table 1).

**Table 1.** Estimates from two univariate animal models applied to investigate sources of phenotypic variation in individual neighbour density in 878 free-living female common terns. All fixed effects, except age, were mean-centred and variance-standardized. Point estimates of fixed and random effects are shown as posterior modes (posterior medians) and [95% CI]. ‘n.s.’ indicates that the interaction was not significant and, hence, was removed from the model in order to better interpret the main effects involved in the interaction. The difference between mean age and delta age (i.e., term 'mean age − delta age') was not fitted as such in the model, but calculated *a posteriori* using the posterior distributions of mean age and delta age

fixed effects	age only		age, corrected for laying date and distance to the wall	
	point estimates	95% **CI**	point estimates	95% **CI**
intercept	1.795 (1.795)	[1.706, 1.874]	1.659 (1.661)	[1.59, 1.737]
mean age	−0.024 (−0.024)	[−0.032, −0.016]	−0.007 (−0.008)	[−0.014, −0.001]
delta age	−0.059 (−0.059)	[−0.078, −0.039]	−0.010 (−0.008)	[−0.013, −0.002]
mean age − delta age	0.033 (0.034)	[0.012, 0.054]	−0.001 (0.001)	[−0.008, 0.009]
mean age × delta age	0.003 (0.003)	[0.001, 0.005]	n.s.	
year	0.015 (0.009)	[−0.086, 0.098]	0.013 (0.021)	[−0.081, 0.107]
colony density	0.037 (0.035)	[−0.043, 0.137]	0.051 (0.048)	[−0.041, 0.143]
island density at breeding	0.671 (0.670)	[0.653, 0.686]	0.608 (0.609)	[0.594, 0.626]
laying date	—	—	0.192 (0.19)	[0.177, 0.201]
distance to wall	—	—	0.084 (0.089)	[0.073, 0.104]

random effects	point estimates	95% **CI**	point estimates	95% **CI**
additive genetic	0.025 (0.027)	[0.012, 0.045]	0.011 (0.013)	[0.004, 0.024]
permanent environment	0.021 (0.020)	[0.003, 0.036]	0.008 (0.009)	[0, 0.017]
year	0.007 (0.009)	[0.003, 0.020]	0.008 (0.010)	[0.003, 0.021]
island identity	0.001 (0.002)	[0, 0.011]	0.000 (0.001)	[0, 0.004]
residual	0.014 (0.014)	[0.008, 0.020]	0.004 (0.004)	[0.002, 0.006]

standardized metrics	point estimates	95% **CI**	point estimates	95% **CI**
repeatability	0.651 (0.639)	[0.522, 0.769]	0.633 (0.590)	[0.409, 0.749]
heritability	0.365 (0.362)	[0.154, 0.584]	0.288 (0.333)	[0.107, 0.625]
evolvability	2.551 (2.451)	[1.685, 3.212]	1.790 (1.703)	[1.030, 2.389]

Interpreting the variance components, common terns showed strong individual differences in their average number of neighbours (repeatability = 65.1%; table 1). Additive genetic effects accounted for 36.5% of the total phenotypic variance, while permanent environmental effects accounted for an additional 28.4% (table 1). The number of neighbours also harboured variance owing to differences among years (9.2%) and islands (0.8%; table 1). Evolvability of this social trait was approximately 2.5% (table 1).

Adding laying date and distance to the nearest wall to the model showed that birds that laid eggs early in the season and nested closer to the wall had, on average, fewer neighbours compared to birds that laid eggs later in the season or in the centre of the island (main effects of laying date and distance to wall; table 1). Importantly, the inclusion of these two variables resulted in a non-significant interaction between the two age effects (within-age and between-age components), which we excluded from the model. The main effects of the two age components were still important, albeit of reduced magnitude, and the difference between them was no longer significant, suggesting that laying date and distance to the wall explain important variation in the selective disappearance process but the observed age effects were not caused solely by older birds having advanced phenology and/or nesting closer to the wall.

### (c) Individual-by-age and genotype-by-age interactions

The random regression model of the number of active neighbours along an age gradient showed evidence for substantial among-individual variation in intercepts and slopes (table 2). Moreover, a DIC model comparison showed that the model with random intercepts and slopes better fitted the data than the model with random intercepts only (DIC of model with random intercepts and slopes = 21 035 and DIC of model with random intercepts only = 21 088). Female common terns, therefore, differed in their average number of neighbours, as well as in the slope of their age-specific decline in the number of neighbours. There was also a significant positive correlation between intercepts and slopes (table 2), indicating a ‘fanning-out’ pattern in individual variation along the age gradient, that is, an increase in individual variance at later ages.

**Table 2.** Estimates from individual and genetic random regression models of neighbour density in 878 free-living female common terns. All fixed effects were mean-centred and variance-standardized. Estimates shown are the among-individual or additive genetic variance in intercepts (*V*_I/A_ intercepts), among-individual or additive genetic variance in slopes (*V*_I/A_ slopes), associated intercepts–slopes covariance (*COV*_I/A_ intercepts–slopes) and correlation (*r*_I/A_ intercepts–slopes), permanent environmental variance in intercepts (*V*_PE_ intercepts), permanent environmental in slopes (*V*_PE_ slopes), associated intercepts–slopes covariance (*COV*_PE_ intercepts–slopes) and correlation (*r*_PE_ intercepts–slopes), among-year variance (*V*_YEAR_), among-island variance (*V*_ISLAND_) and heterogeneous residual variances (*V*_RESIDUAL_). Point estimates of fixed and random effects are shown as posterior modes (posterior medians) and [95% CIs].

fixed effects	individual model		genetic model	
	point estimates	95% **CI**	point estimates	95% **CI**
intercept	1.605 (1.586)	[1.517, 1.649]	1.595 (1.599)	[1.529, 1.665]
age	−0.127 (−0.136)	[−0.164, −0.11]	−0.133 (−0.128)	[−0.157, −0.096]
year	0.006 (−0.002)	[−0.101, 0.085]	0.016 (0)	[−0.095, 0.094]
colony density	0.057 (0.04)	[−0.05, 0.134]	0.047 (0.049)	[−0.033, 0.154]
island density at breeding	0.674 (0.672)	[0.652, 0.689]	0.667 (0.669)	[0.653, 0.688]

random effects	point estimates	95% **CI**	point estimates	95% **CI**
*V*_I/A_ intercepts	0.046 (0.048)	[0.039, 0.061]	0.031 (0.029)	[0.015, 0.04]
*V*_I/A_ slopes	0.020 (0.024)	[0.014, 0.035]	0.019 (0.02)	[0.011, 0.029]
*COV*_I/A_ intercepts-slopes	0.013 (0.013)	[0.006, 0.023]	0.014 (0.012)	[0.003, 0.019]
*r*_I/A_ intercepts–slopes	0.417 (0.401)	[0.239, 0.573]	0.500 (0.489)	[0.164, 0.766]
*V*_PE_ intercepts	—	—	0.019 (0.019)	[0.006, 0.031]
*V*_PE_ slopes	—	—	0.000 (0.004)	[0, 0.013]
*COV*_PE_ intercepts–slopes	—	—	0.000 (0)	[−0.004, 0.010]
*r*_PE_ intercepts–slopes	—	—	0.135 (0.103)	[−0.481, 0.692]
*V*_YEAR_	0.007 (0.010)	[0.003, 0.023]	0.011 (0.012)	[0.003, 0.025]
*V*_ISLAND_	0.001 (0.001)	[0, 0.008]	0.001 (0.001)	[0, 0.004]
*V*_RESIDUAL_ [1]	0.003 (0.004)	[0.001, 0.012]	0.003 (0.004)	[0.002, 0.013]
*V*_RESIDUAL_ [2]	0.002 (0.004)	[0.001, 0.009]	0.002 (0.004)	[0.001, 0.01]
*V*_RESIDUAL_ [3]	0.007 (0.008)	[0.001, 0.02]	0.006 (0.007)	[0.001, 0.018]
*V*_RESIDUAL_ [4]	0.039 (0.037)	[0.02, 0.052]	0.034 (0.036)	[0.02, 0.053]
*V*_RESIDUAL_ [5]	0.004 (0.005)	[0.001, 0.012]	0.004 (0.006)	[0.001, 0.011]

**Figure 3.**
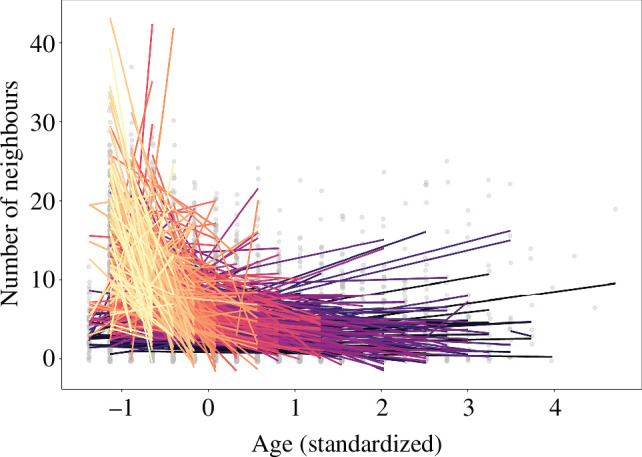
Predictions from the animal random regression model of additive genetic variation in neighbour density as females grow older (genotype-by-age interaction). Each coloured line represents a single individual female common tern. Colours are ordered from lightest to darkest to facilitate visualization of individual slopes. Raw data points are jittered for illustration purposes and shown in grey in the background. Age was mean-centred and variance-standardized.

DIC model comparison showed that the animal model that split phenotypic variation into the additive genetic and permanent environmental components of individual plasticity better fitted the data than the model with random intercepts and slopes at the individual level (DIC of model with random intercepts and slopes at additive genetic and permanent environmental levels = 21 011 and DIC of model with random intercepts and slopes at individual level = 21 035). When partitioning the among-individual variation in reaction norms into additive genetic and permanent environmental effects, we found heritable differences only (i.e. strong support for G × Age but no support for PE × Age; table 2 and figure 3). Covariances and correlations between intercepts and slopes at the genetic level were significantly positive, but there was no such pattern at the permanent environmental level (table 2).

## 4. Discussion

Longitudinal studies of various vertebrate populations increasingly provide evidence for age specificity of phenotypic traits (reviewed in [15]), including, in more recent years, in social behaviour (e.g. [20] in a bird species, reviewed in [18]; Woodman *et al*. [19] and Firth *et al*. [76]). In female common terns, we observed a significant decline in the number of active neighbours at the time of clutch initiation, in line with previous findings of older animals having reduced sociality (e.g. [18,20,34,40–44], but see [39]). Although this decline in social behaviour with age has been found across different taxonomic groups, it is important to note that a large number of studies focused on humans and non-human group-living mammals—on primates in particular [18]. Additionally, the vast majority of studies focused on sociality measured as associations between individuals (e.g. connectivity and affiliative interactions), applying methodological tools such as social network analysis [77–79].

In our population, breeding phenology is strongly correlated with individual age (*r* = −0.48, 95% CI [−0.72, −0.14]; also see [48]), with older females breeding on average earlier in the year than younger ones. In this study, we found an important effect of egg-laying date on our social trait, with an early start of breeding being associated with having fewer neighbours compared to when egg-laying started later in the season (table 1). In principle, the observed association between age and social behaviour could therefore have been indirectly mediated by breeding phenology. Similarly, previous work in our colony suggested that older birds breed closer to the island edges than younger birds [57], which we corroborated in our study (electronic supplementary material, table S3). Unlike in populations breeding in natural habitat, where safety from predators is higher in the centre, common terns breeding closer to the island edges at the Banter See site may be safer from aerial predators such as crows (that predate eggs) or owls (that predate chicks), which would have an easier line of attack in the centre of our (small: 10.7 × 4.6 m) islands, given the 60 cm-high walls that were built to protect these islands against flooding and rats. Breeding at the island edges necessarily leads to having fewer neighbours within a 2 m radius, such that the observed association between age and social behaviour could have been indirectly mediated by distance to the edge as well. Despite both changing with age and explaining a substantial amount of variation in our social trait, adding laying date and distance to the island edge to the model did not cause our estimates for our two age components to become unimportant (the main effects of average and delta age were reduced but remained statistically significant; table 1). As such, we suggest that behaviour—additional to the timing of breeding and spatial nesting distribution within the colony—will be responsible for part of the social ageing pattern we observed. Active within-colony territory defence could represent such behaviour and may be beneficial to maintain a sufficiently large landing spot for parents carrying fish to provision their chicks during the rearing period (given that chicks are at risk of kleptoparasites when parents transfer their fish to them) or to lower infection risks in the case of disease outbreaks (see Siracusa *et al*. [80] and Albery *et al*. [81] (both this issue) for two studies on infectious disease risk and group-living behaviour).

The age-specific decline in the number of neighbours was significantly stronger among than within individuals, indicating selective disappearance of individuals with a large number of neighbours from the breeding population. Given that this effect became non-significant when we corrected for the laying date and distance to the colony edge, the latter of which itself showed signs of selective disappearance (electronic supplementary material, table S3), survival selection may operate on phenology (also see [82]) or via predation on birds nesting more centrally. Alternatively, the ability to nest early and/or close to the colony edge may not be causally related to survival but instead reflect a quality aspect that we do not yet quite understand. If related to social dominance, our results contrast with those obtained in a study on jackdaws, where dominant (not subordinate) birds disappeared from the colony at a younger age compared to subordinate ones [20]. They also contrast with results of two studies in rhesus macaques (*Macaca mulatta*) and red deer that found no evidence for selective disappearance with respect to social behaviour ([34,41], but see [40]). Whether this variation in effects among species is underpinned by variation in the strength of competition or other factors is currently unknown, but warrants further empirical attention.

In addition to social ageing and selective disappearance of birds with many neighbours, we found strong support for individual variation in the rate of social ageing [38], as well as, remarkably, for heritable differences to underpin most of this variation (table 2). Collective evidence regarding a genetic basis underlying rates of ageing in natural populations is still mixed and mostly focused on fitness-related traits (reviewed in [36,37]), with three out of five studies having documented an increase in genetic variation with age [33,38], while the two other studies only reported individual, but not genetic, variation in ageing rates (although statistical power could have hampered those analyses [27,28]). Our study differentiates from these previous studies in that it reports on the quantitative genetics of a behavioural trait, a part of the phenotype that has so far remained relatively understudied [83]. Our results, however, fit well with those from a study by Class *et al*. [84], which provided support for G × Age in exploratory behaviour in wild great tits. In the presence of substantial G × Age and a positive intercept–slope correlation, suggesting a ‘fanning-out’ pattern, evolutionary changes in social behaviour will strongly depend on selection acting on later age classes (when additive genetic variance is at its highest), whereas selection on social behaviour at younger ages (when additive genetic variance is at its lowest) would have a reduced evolutionary impact.

Overall, our study provides strong evidence for social ageing in a long-lived, colonially breeding seabird species and, hence, support for the recently postulated role of social behaviour as one of the aspects or drivers of the ageing phenotype [18]. At the same time, it raises the question of whether such social ageing patterns encompass other social traits as well and/or whether they occur at other stages of the annual cycle. Additionally, our finding of heritable differences in individual variation in social ageing is novel and gives rise to further research questions related to the genetic control of ageing and the consequences of genotype-by-age interactions for the evolution of the overall ageing phenotype, especially given our finding of selective disappearance of birds experiencing a more social environment.

## Data Availability

All materials, including the relevant data and R-code for the different analyses, are available from the Dryad database [85]. Supplementary material is available online [86].
